# Moving towards sustainable food systems: A review of Indian food policy budgets

**DOI:** 10.1016/j.gfs.2020.100462

**Published:** 2021-03

**Authors:** Kerry Ann Brown, Nikhil Srinivasapura Venkateshmurthy, Cherry Law, Francesca Harris, Suneetha Kadiyala, Bhavani Shankar, Sailesh Mohan, Dorairaj Prabhakaran, Cécile Knai

**Affiliations:** a Faculty of Public Health & Policy, London School of Hygiene & Tropical Medicine, London, UK; b Centre for Chronic Disease Control, New Delhi, India; c Public Health Foundation of India, Gurgaon, India; d Department of Population Health, London School of Hygiene & Tropical Medicine, London, UK; e Centre on Climate Change and Planetary Health, London School of Hygiene & Tropical, London, UK; f Institute for Sustainable Food, University of Sheffield, Sheffield, UK

**Keywords:** Sustainable development goals, India, Food system, Nutrition, Environment, Equity

## Abstract

Moving towards sustainable food systems is a complex problem, which requires high level co-ordination, coherence, and integration of national food policy. The aim of this study is to explore where environmental sustainability is integrated into national food policy in India. A scoping review of food policies was conducted, and findings mapped to ministerial responsibility, estimated budget allocation, and relevant Sustainable Development Goals. Fifty-two policies were identified, under the responsibility of 10 ministries, and with relevance to six Sustainable Development Goals. Content analysis identified references to environmental sustainability were concentrated in policies with the smallest budgetary allocation. Resources together with political will are required to integrate environmental sustainability into food policies and avoid conflicts with more well-established health, societal, and economic priorities.

## Highlights

• Environmental sustainability is not consistently integrated across Indian food policy.
• Food policy in India, like most countries, is disparate and fragmented.
• Food production policies are the most likely to refer to environmental sustainability.
• Environmental sustainability is infrequently referenced in food consumption policies.
• The policies that prioritised environmental sustainability were the ones that received the least funding.

## 1 Introduction

Change is required in how we govern our food systems, as they do not currently provide universal health, wealth, or environmental sustainability to communities around the world (Sachs et al., 2019; TWI2050, 2018). Globally, two billion people experience moderate to severe food insecurity, and obesity contributes four million deaths (FAO, IFAD, UNICEF, WFP and WHO, 2019). Inequalities in food security will increase with climate change. Populations with a higher prevalence of hunger and malnutrition are more likely to be impacted by the reduced food availability (lower yields), food accessibility (higher prices), and ultimately food utility (unclean water) associated with warmer temperatures, irregular rainfalls, and extreme weather events (Wheeler and von Braun, 2013). This is exacerbated by agricultural production, which itself contributes to climate change. Food production accounts for approximately a quarter of global greenhouse gas emissions (via energy use, rice methane, ruminant enteric fermentation etc.) (World Resources Institute, 2019). Agriculture is also a key driver of changes to water use and land use (e.g., from 1961 to 2017, the production of cereal crops increased by 240% due to land expansion and increasing yields, and the use of irrigation water almost doubled (Shukla et al., 2019).

Governments around the world have the ability to transform our food systems, yet this will require co-ordinated, coherent, and integrated action (Weitz et al., 2017). Food reaches many areas of our lives from health (nutrition), to society (culture), economics (jobs), and the environment (biodiversity). All of the policies that govern these areas, however, continue to be developed and implemented in silos (International Council for Science ICSU, 2017). Government ministries might share information about on-going initiatives, and at times design policies with common objectives. Rarely though will policies be integrated, so that it clear how each policy fits into an overarching aim, such as moving towards sustainable food systems (Cejudo and Michel, 2017). This means policy is often inefficient, with priorities in one area potentially in conflict, negating, or leading to unintended negative consequences in another area (OECD, 2013; Rasul, 2016).

Conflicts between existing policies are context specific to different countries and regions, dependent upon political priorities that may have changed over time (Thow et al., 2016). For example, Indian food policy has traditionally supported food production, agricultural livelihoods, trade, and food security. Agriculture contributed over 15% to India's Gross Domestic Product in 2019 (The World Bank, OECD, 2020), and the country is now one of the largest producers of agricultural outputs in the world (OECD-FAO, 2020). A comprehensive package of food security safety nets also exists in India, supported by a constitutional ‘right to food’ (Narayanan and Gerber, 2017). These safety nets are necessary and becoming more targeted to nutrition security, as India continues to tackle the burden of diseases associated with poor quality diets (e.g., a stunting prevalence of over 30% in children under five, and over half of pregnant women recognised as anaemic, as well as increasingly rates of overweight and obesity and diabetes mellitus type 2) (Shankar et al., 2017; Swaminathan et al., 2019; FAO, IFAD, UNICEF, WFP and WHO, 2020; India State-Level Disease Burden Initiative Malnutrition Collaborators et al., 2019).

More recently, there has been a concern over environmental resources in India, which are becoming increasingly vulnerable to climate change, climate variability and climate uncertainty (Hinz et al., 2020). In particular, the sustainability of fresh water. Over 50% of Indian food production regions have been impacted by irregular seasonal rains, leading to an overuse of groundwater, depleting aquifers, and competition between agricultural and drinking water supplies (World Bank Group, 2018). Choosing to prioritise health, social, or even wider economic development, at the expense of environmental consequences has put at risk India's ability to continue to expand agricultural production, support farmer livelihoods, increase international and domestic trade, and ensure food security for a growing population (projected to be 1.6 billion by 2045, with current fertility rates) (United Nations, 2019).

How to integrate environmental sustainability into food policy, alongside existing priorities is a challenge (Biermann et al., 2017; Sachs et al., 2019). First, it is difficult to identify all policies that are relevant to food systems. The concept of food systems is complex. At the most basic level, they represent the food supply chain (production, processing, distribution, retail, consumption and disposal or waste of food) (Parsons et al., 2019a). The relationship between these activities and wider systems is intricate and dynamic, and can differ dependent on whether food is being viewed as a tradable commodity (economic system), a human right (social system), a requirement (health system), or part of the local environment (ecological system) (Garnet, 2013; Parsons et al., 2019a; Davies, 2020) (see Fig. 2 for a representation of the many different activities and overlapping elements of food systems). In addition, parts of the food system can be opaque and do not always fall under the governance of national or international governments (e.g., corruption and food fraud) (Lord et al., 2017). It therefore, becomes difficult to identify and integrate a myriad of policies, which in/directly impact the food system, and impossible to implement effective food policy in areas that lack transparency (Global Panel on Agriculture & Food Systems for Nutrition, 2016).

**Fig. 1:**
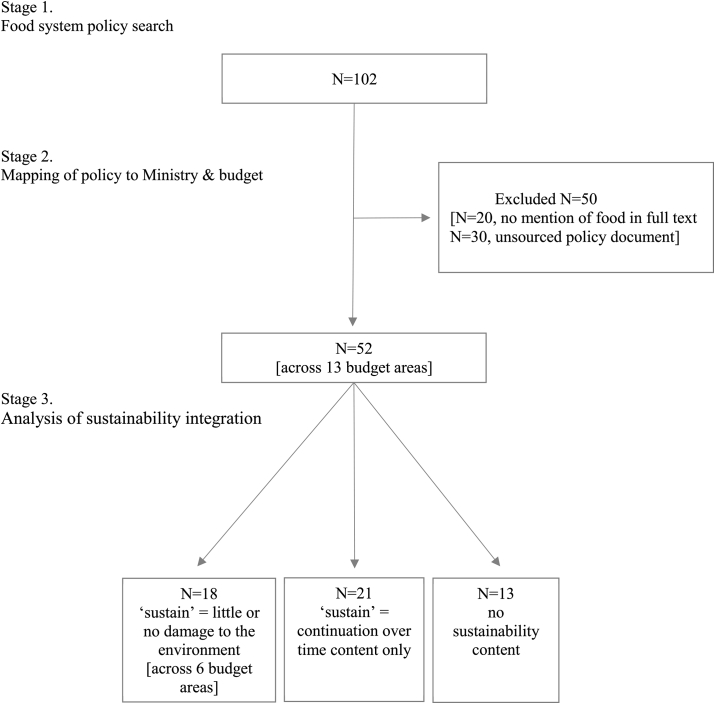
Summary of the scoping review process to a) identify policies relevant to the Indian food system, b) identify the resources allocated to these policies, c) explore the integration of environmental sustainability in Indian food policy.

**Fig. 2:**
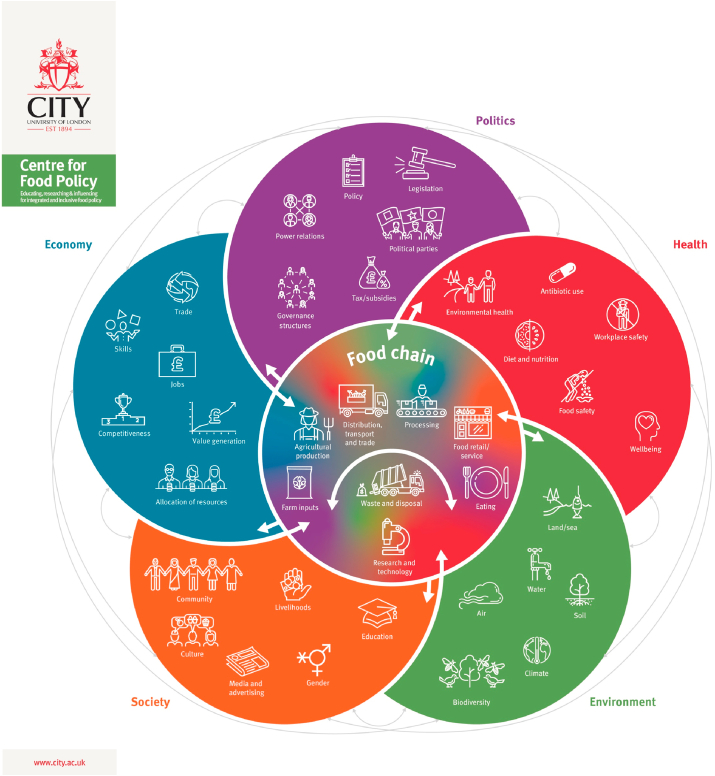
Representation of the food supply chain and connections to wider overlapping systems (colour). Reproduced with permission by Parsons K, Hawkes C, Wells R. Brief 2. What is the food system? A Food policy perspective. In: Rethinking Food Policy: A Fresh Approach to Policy and Practice. London: Centre for Food Policy; 2019. (For interpretation of the references to colour in this figure legend, the reader is referred to the Web version of this article.)

Second, sustainability is multi-faceted and ill-defined (Béné et al., 2019). In its most holistic form, sustainability represents three dimensions: to sustain the health of populations, society as a whole, and the health of the planet (Friel et al., 2008). The latter is the most commonly referred to sustainability dimension and the main focus of the current study. Environmental factors, such as staying within planetary boundaries on climate change, biodiversity, Phosphorus and Nitrogen cycles, freshwater use, ocean acidification, land use (deforestation, removal of peat lands) etc. are all relevant to the food system (Rockström et al., 2009). These factors are dynamic, for example, water use will differ dependent on the geographic location of food production, time (e.g., season), and production methods use (e.g., irrigated vs. rainfed crops) (Kayatz et al., 2019). Furthermore, improvements on one environmental factor may not always translate to universal environmental benefits, as one intervention might promote efficient water use, yet, be contributing to carbon dioxide, methane or nitrous oxide greenhouse gas emissions (Poore and Nemecek, 2018). This makes it difficult to define environmental sustainability, which in turn complicates how it can be balanced with other priorities and integrated into food policy (Béné et al., 2019).

The UN 2030 Sustainable Development Goals (SDG) provide one way to integrate food policy. The SDG comprise 17 goals, 169 targets and 232 indicators agreed by national governments around the world. These goals integrate the three dimensions of sustainability to encourage a holistic concept of sustainable development, whereby the concepts of health, society, and the environment are valued alongside economic markers of progress, such as Gross Domestic Product (Griggs et al., 2013). Several SDG are relevant to the food system, from those designed to tackle hunger, good health and well-being, to those targeting affordable and clean energy, responsible consumption and production, or climate action (SDG 2, SDG 3, SDG 7, SDG 12, SDG 13 respectively). Global progress on the SDG has been slow and one of the reasons cited for this is the continuing competition and tensions between policies which prioritise trade or different dimensions of sustainability (Lyytimäki and Rosenström, 2008; Pintér et al., 2012; ICSU, 2018; United Nations Department of Economic and Social Affairs, 2019). This is illustrated by the health orientated SDG 3 good health and well-being, which is considered to be in conflict with the environmental focused SDG 12 responsible consumption and production. This is because as a country's health improves, it is continuing to do so by generating wealth through routes which are not environmentally sustainable (Pradyumna, 2018; Pradhan et al., 2017). Another example is the SDG 7 affordable and clean energy, which has been cited as a risk to fulfilling the food security SDG 2 zero hunger where agricultural land is used for bioenergy production (Nilsson et al., 2016).

The Indian government is committed to the sustainability agenda, signing up to both the SDG in 2015, and the Paris Climate Agreement in 2016 (the latter pledging to limit the impact of climate change by reducing green-house gas emissions and minimising global temperature rises). Political infrastructure has also been dedicated the responsibility of co-ordinating the national response to SDG. The cross-cabinet body of the National Institute for Transforming India (NITI Aayog, formerly the Planning Commission) facilitates the gathering and sharing of information between states and ministries (policy co-ordination), with the aim to promote policy coherence and the integration of SDG throughout state and national policy. One of the first activities of NITI Aayog included mapping policies to SDG to identify relevant targets and indicators for measuring SDG progress. This produced an SDG mapping document (Government of India, 2019a), which included several national programme. It has not been clear however, which of these programme are relevant to the food system, how large or influential these policies are, nor the degree to which environmental sustainability has already been integrated across food policy in India. The aim of this study is to explore the degree to which environmental sustainability is integrated into existing food policy in India by a) completing a scoping review of policies relevant to the food system, b) exploring the infrastructure and resources (Ministries and estimated budget) responsible for delivering these policies c) considering whether the concept of environmental sustainability is integrated across the policies identified, and how food policies are distributed across different SDG (which prioritise different dimensions of sustainability and priorities for sustainable development).

## 2 Methods

The review was conducted in three stages (Fig. 1). Stage 1 identified Indian food system policies. Stage 2 mapped food system policies to a Ministry and budget resources responsible for delivering these policies. Stage 3 explored if environmental sustainability was integrated across the policies by performing qualitative content analysis of the policy documents and using prior work by NITI Aayog to present which food policies targeted which SDG. This is a scoping review of policy documents and as such no systematic review protocol has been registered. Methods have been reported in line with those established in the PRISMA extension for scoping review guidelines (Tricco et al., 2018).

### 2.1 Stage 1 food system policy search (Table 1)

#### 2.1.1 Information sources

The main review was conducted between Oct 2017 and Mar 2018 and updated with current budget information in Apr 2020. Government of India, Ministry and Departmental websites were searched for the word ‘food’ to identify which national policies could be relevant to the Indian food system. This was conducted using the ‘national portal of India’ search function and the 26 ‘topic buttons’, both of which are on the Government of India homepage (Government of India National Portal, 2020). These search functions led to the manual search of 15 individual Ministry websites. The Ministry of Finance and Ministry of Defence websites were excluded from the search (see section 2.1.2).

#### 2.1.2 Eligibility criteria

Due to the available research resources, the policy document search was limited to centrally sponsored or central sector schemes i.e., policies at the national level. India operates a quasi-federal government with different states/union territories retaining a degree of autonomy over the allocation and utilisation of financial funds; however, no policies were reviewed at the Indian state or union territory level in the current study. This would involve reviewing policies across 28 states and eight union territories; beyond the scope of the resources available. This is why the Ministry of Finance and Ministry of Defence websites were excluded. The majority of the budget in these Ministries does not relate to centrally sponsored or central sector schemes, with approximately a quarter of the total Government of India budget allocated to national debt interest repayments and national defence spending. (Government of India, 2018a; Government of India, 2019c, Government of India, 2019b; Government of India, 2020a). Excluding these websites and policies was also designed to prevented confusion between scheme revenue and expenditure (e.g., related to the General Services Tax) or possible duplication of schemes (e.g., procurement of military foodstuffs via the Public Distribution System). Only documents in English were reviewed. This did not exclude any central policies as all national policies are mandated to be available in English (as an additional language to Hindi). A broad working definition of policy (“a set of ideas or plans that is used as a basis for making decisions, especially in politics, economics, or business” (Collins Collins Free Online Dictionary, 2020) was used to include a range of policy documents: missions, acts, programme and schemes.

#### 2.1.3 Full text screening

Full text policy documents were sourced for all the policies identified from the website searches. Where no policy document was available, substitutes were used that included policy goals and objectives, such as the legal publication of a policy in the Gazette of India (a public journal and authorised legal document of the Government of India), or a PDF created from the policy webpage. Policies were excluded where no policy document or substitute was available. All documents were content searched for the word ‘food’. Food did not have to be the direct target for every policy. The policy did, however, need to be relevant to at least one part of the food supply chain or wider system (as represented in Fig. 2). Policies were excluded if there was no mention of ‘food’ in the full text document.

### 2.2 Stage 2 mapping of policy to ministry & budget

#### 2.2.1 Allocation of ministry responsibility & budget in/exclusion

The Government of India's estimated expenditure budget of 2018–2019 was used to allocate Ministerial responsibility and funding resources for each policy (Government of India, 2018b). The review was updated by tracking the 2019–2020 and 2020–2021 estimated expenditure budgets i.e., to verify whether each policy was still active and identify the latest funding allocation (Government of India, 2019b; Government of India, 2020b). These budget documents list individual policies and also aggregate individual policies into larger budget areas e.g., the National Livestock Mission is listed as part of a larger dairy (White Revolution) budget area in the Ministry of Fisheries, Animal Husbandry and Dairying.

The majority of food system policies fell under a clear budget area. The exception was the National Action Programme for Climate Change. This is a cross-ministerial policy, where different sub-policies came under the budget of different Ministries; therefore, funds for each sub-policy had to be summed manually to create a climate change budget area. Care was taken to avoid duplication/double counting of funds e.g., the National Mission for Sustainable Agriculture is a sub-policy of the National Action Programme for Climate Change, yet a number of National Mission of Sustainable Agriculture policies were listed individually under the agricultural production (Green Revolution) budget area. These were subtracted from the agricultural production budget and manually added to the climate budget.

Budgets for the food system policies identified were calculated by subtracting excluded policies (in line with section 2.1.3) e.g., the social assistance budget included three pension schemes and the Annapurna Scheme (the latter, a scheme which improves food security in older people). None of the pension scheme policy documents mentioned food. These were excluded and their budgets subtracted from the total budget, which meant, in effect, the Annapurna Scheme represented the social assistance budget area.

Policies were excluded if they fell within a budget area of less than ₹500 crores (equivalent of 5 billion rupees or approximately US$65.5 million). This exclusion criterion limited the number of policies reviewed to ensure the review was feasible, within resource constraints, whilst capturing the main policies and budget areas relevant to the food system. Individual policies with a budget less than ₹500 crores were included if they were aggregated to a budget area over ₹500 crores.

### 2.3 Stage 3 analysis of sustainability integration

#### 2.3.1 Content analysis for environmental sustainability

Policy documents considered relevant to the Indian food system were searched for sustainability content. This provided a means to identify if the concept of sustainability was integrated into the framing of each policy. Each policy document was searched for the words ‘sustainable’ and ‘sustainability’. Policies which referred to sustainability in terms of little or no damage to the environment were considered sustainable food system relevant policies. Findings were recorded in a coding template developed to differentiate between two dictionary definitions of sustainability: i) ‘sustain-able/-ability’ meaning to continue over time, ii) ‘sustain-able/-ability’ meaning little or no damage to the environment (Collins Collins Free Online Dictionary, 2020). This provided a way to record both the content and meaning of sustainability, which is in line with basic content analysis, and allowed the distinction between environmental sustainability over other sustainability concepts (Braun and Clarke, 2006).

#### 2.3.2 Distribution of food policies over SDG

Results of the review are presented by mapping food policies onto one of the 17 SDG. Although all SDG are interrelated, each SDG has a different focus and alignment with one or other SDG can represent priority areas for different dimensions of sustainability. Mapping was completed by adapting the NITI Aayog Government of India framework which maps central sector schemes by SDG targets (Government of India, 2019a). The majority of food system policies reviewed in this study were not included in the Government of India document, therefore only the larger budget areas have been mapped onto SDG. Findings are presented and interpreted by exploring the distribution of food system policies across the SDG, in terms of their frequency (number of policies) and resources provided (Ministerial responsibility and budgetary allocation).

## 3 Results

**Fig. 3:**
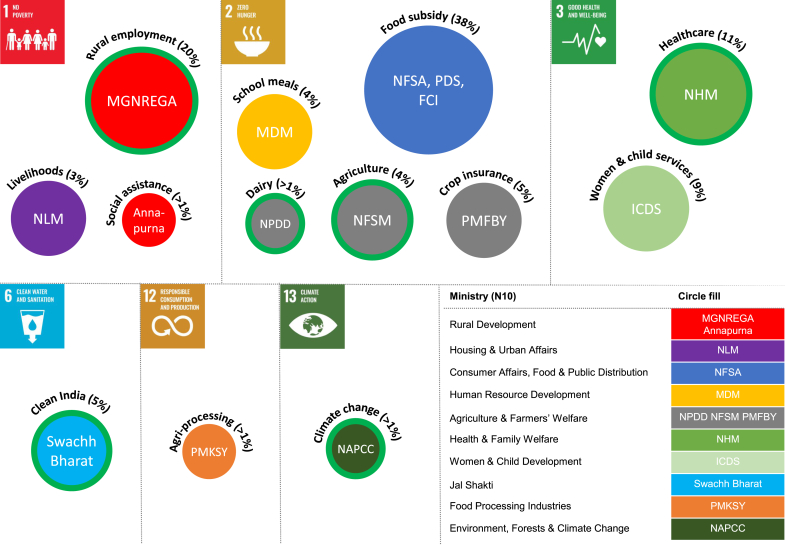
Policy areas relevant to the Indian food system, mapped by Sustainable Development Goal and Ministry. Size of circle represents the percentage of a calculated food policy budget using the 2020–2021 Government of India estimated budget (total INR 30,1694 crores/USD 39.5 billion). Indicative policies shown within circles. Green outlines indicate areas which included sustainable food policies (colour). (For interpretation of the references to colour in this figure legend, the reader is referred to the Web version of this article.)

A total of 52 food system relevant policies were reviewed. One hundred and two policies were identified from Government of India websites, of which fifty were excluded (food was not included in the full text policy document or a policy document/substitute could not be sourced, Fig. 2). The 52 policies came under 13 budget areas and the responsibility of 10 ministries (Table 2/Fig. 3). The total funds allocated to the 52 policies were ₹30, 16, 94 crores in 2020–2021 (US$39.5 billion), which is 10% of the total 2020–2021 Government of India estimated budget (₹30, 42, 230 crores/US$0.4 trillion). Findings below present the distribution of food system policies across SDG, in terms of their frequency (number of policies) and resources provided (Ministerial responsibility and % of the 2020–2021 ₹30, 16, 94 crores budgetary allocation), before exploring the degree sustainability was integrated across the policies identified.

**Table 1:** Policy document search strategy and in/exclusion criteria.

Information sources	Eligibility criteria	Search term
Government of India National Portal of India Government of India Topic Buttons Government of India Ministry and Department websites	English Language National centrally sponsored/central sector scheme Policy definition including missions, acts, programme, and schemes Policy present in the Government of India budget Policy within a budget area over ₹500 crores (₹5 billion/ US$65.5 billion) Policy document or substitute available (Gazette of India or detailed policy webpage)	Food

**Table 2:** National Government of India (GoI) food policies identified in scoping review, presented by relevant Sustainable Development Goal (SDG) and allocated budgets calculated from the estimated budgets over three budget cycles from 2018 to 2020 (figures in Crores of Indian Rupees and rounded to nearest whole number). Bold text denotes where policy documentation refers to environmental sustainability.

Relevant SDG (N6)	Budget area (N13)	Food policy (N52)	Ministry (N10)	2018–2019 food relevant policy budget in ₹ crores (% of total)		2019–2020 food relevant policy budget in ₹ crores (% of total)		2020–2021 food relevant policy budget in ₹ crores (% of total)	
1 No poverty	Rural employment	**MGNREGA**	Rural Development	**55,000**	**(16.2%)**	**60,000**	**(16%)**	**61,500**	**(20%)**
	Social assistance	NSAP; Annapurna Scheme		78	(<1%)	63	(<1%)	63	(<1%)
	Livelihoods	DAY-NULM; NRLM	Housing & Urban Affairs	6060	(1.8%)	9774	(2.7%)	10,005	(3.3%)
2 Zero hunger	Food subsidy	PDS; NFSA; Sugar subsidy AAY/PDS; Decentralised procurement of food grains under NFSA; Subsidy to FCI under NFSA	Consumer Affairs, Food & Public Distribution	169,323	(50%)	184,220	(50.5%)	115,570	(38.3%)
	Agricultural production	**NFSM**; **NMH**; NMOOP; RKVY; **PKVY**; **NPOF**; **SMSP**; **SMPPPQ**; **NMAET**; SMAM; ISEACS; ISAC; ISAM	Agriculture & Farmers' Welfare	**12,684**	**(3.7%)**	**11,586**	**(3.2%)**	**12,441**	**(4.1%)**
	Crop insurance	PMFBY		13,000	(3.8%)	14,000	(3.8%)	15,695	(5.2%)
	Dairy	**NPDD**; LCISS; NLM		**724**	**(<1%)**	**955**	**(<1%)**	**770**	**(<1%)**
	School meals	Mid-Day Meal in Schools	Human Resource Development	10,500	(3.1%)	11,000	(3%)	11,000	(3.7%)
3 Good health & well-being	Healthcare	NHM; NUHM; NRHM; TCP; **National AYUSH Mission**	Health & Family Welfare	**30,634**	**(9%)**	**33,651**	**(9.2%)**	**34,105**	**(11.3%)**
	Women and child services	Anganwadi services core ICDS; NNM; PMMVY/MBS; RESEAG-SABLA; NCS; CPS	Women & Child Development	23,088	(6.8%)	27,584	(7.6%)	28,557	(9.5%)
6 Clean water & sanitation	Clean India	**Swachh Bharat Mission (Gramin)**	Jal Shakti	**15,343**	**(4.5%)**	**9994**	**(2.7%)**	**9994**	**(3.3%)**
12 Responsible production & consumption	Agri-processing	SAMPADA; PMKSY (Mega food parks)	Food Processing Industries	1313	(<1%)	1101	(<1%)	1081	(<1%)
13 Climate action	Climate change*	**NAPCC**; NAF; **NMGI**; **NWM**; **NMSHE**; **NMSKCC**; **NMSA** (NBM; NPSHF; **NPAF**)	Environment, Forests & Climate Change	**1424**	**(<1%)**	**1164**	**(<1%)**	**913**	**(<1%)**
TOTALS				339,171		365,092		301,694	

*The climate budget has been estimated using individual policies and includes internal budgeting/EAP components. The National Mission on Sustainable Agriculture (NMSA) and its sub-policies have been included under climate they are a sub-policy of the National Action Plan on Climate Change (NAPCC).

**Key:** AAY: Antyodaya Anna Yojana; AYUSH: Ayurveda, Siddha and Unani & Homeopathy; CPS: Child Protection Services; DAY-NULM: Deendayal Antyodaya Yojana-National Urban Livelihood Mission; FCI: Food Corporation of India; ICDS: Integrated Child Development Services scheme; ISAC: Integrated Scheme on Agricultural Cooperation; ISAM: Integrated Scheme for Agricultural Marketing; ISEACS: Integrated Scheme on Agriculture Census and Statistics; LCISS: Livestock Census and Integrated Sample Survey; MGNREGA: Mahatma Gandhi National Rural Employment Guarantee Programme; Mid-day Meals: National Programme of Mid-Day meal in Schools; NAPCC: National Action Programme on Climate Change; NBM (NMSA): National Bamboo Mission; NCS: National Creche Scheme; NFSA: National Food Security Act; NFSM: National Food Security Mission; NHM: National Health Mission; NLM: National Livestock Mission; NLM-Ajeevika: National Livelihood Mission-Ajeevika; NMAET: National Mission on Agriculture Extension and Technology; NMH: NMGI: National Mission for a Green India; NMH: National Mission on Horticulture; NMOOP: National Mission on Oilseeds and Oil Palm; NMSA: National Mission on Sustainable Agriculture; NNM: National Nutrition Mission/Prime Minister's Overarching Scheme for Holistic Nourishment (POSHAN-Abhiyaan); NPAF (NMSA): National Project on Agroforestry; NPDD: National Programme for Dairy Development; NPSHF (NMSA): National Project on Management of Soil Health and Fertility; NRLM: National Rural Livelihood Mission; NRHM: National Rural Health Mission; NSAP: National Social Assistance Program; NUHM: National Urban Health Mission; PDS: Public Distribution Scheme; PKVY: Paramparagat Krishi Vikas Yojana; PMFBY: Pradhan Mantri Fasal Bima Yojana; PMKSY: Pradhan Mantri Kisan Sampada Yojana; PMMVY/MBS: Pradhan Mantri Matru Vandana Yojana/Maternity Benefit Programme; RESEAG-SABLA: Rajiv Gandhi Scheme for Empowerment of Adolescent Girls; RKVY: Rashtriya Krishi Vikas Yojana; SAMPADA: Scheme for Agro-Marine Processing and Development of Agro-processing clusters; SMAM: Sub-mission on Agricultural Mechanization; SMPPPQ: Sub-Mission on Plant Protection and Plant Quarantine; SMSP: Sub-Mission on Seeds and Planting Material; TCP: Tertiary Care Programme.

### 3.1 Food policy & society (SDG 1 no poverty)

Five of the 52 policies and almost a quarter of the budget are relevant to SDG 1 no poverty (₹71,568 crores/US$9 billion). This included the three budget areas of rural employment (via the Mahatma Gandhi National Rural Employment Guarantee Programme), social assistance (via the Annapurna food security scheme), and livelihoods (via support for food production and food service livelihoods, as well improving food security of the homeless). These social policies are designed to reduce poverty and protect vulnerable populations across the food system: relevant to food production, food retail/service, and food consumption activities.

### 3.2 Food policy & health (SDG 2 zero hunger, SDG 3 good health & well-being, SDG 6 clean water & sanitation)

The majority of the policies identified targeted health and the SDG of zero hunger (SDG 2), good health & well-being (SDG 3), and clean water & sanitation (SDG 6). These represented over two thirds of the 52 policies and 75% of the budget (35/52 policies, totalling ₹2,28,132 crores/US$29.8 billion). Swachh Bharat or Clean India policies to eliminate open defaecation and improve waste management were identified as relevant to SDG 6 clean water & sanitation and included due to their food safety relevance. The remainder of the SDG 2 and SDG 3 policies were associated with food security in its broadest sense: designed to increase the availability, access, and utility of food.

Five policy budget areas, 23/52 individual policies, and 52% of the budget was mapped to SDG 2 zero hunger. This included food subsidy policies (e.g., the Public Distribution System); agricultural production policies (e.g., National Mission on Horticulture); school meal policies (Mid-Day Meal scheme); dairy policies (e.g., National Livestock Mission); and crop insurance policies (Pradhan Mantri Fasal Bima Yojana). The latter crop insurance scheme was mapped to SDG 2 in the NITI Aayog Government of India mapping exercise (Government of India, 2019a), however, it is recognised that this policy is also relevant to SDG 1 no poverty, as farmer livelihoods clearly link SDG 1 & 2. The largest budget was seen for food subsidies. The Ministry of Consumer Affairs, Food & Public Distribution received 38% of the budget reviewed (₹1,15,570 crores/US$15 billion), which was the equivalent of 4% of the total Government of India estimated budget for 2020–2021 (including all policies, food and non-food).

Policies related to SDG 3 good health and well-being also accounted for substantial sums of the food system relevant policies identified. Healthcare policies (e.g., the National Health Mission), as well as women and children policies (including the National Nutrition Mission) received 20% of the budget (₹62,662 crores/US$8.2 billion). These two policies referenced a wide range of programme relevant to food and health, and the need for co-ordination between different (government and non-government) sectors. For example, one of the National Health Mission's guiding principles was to ‘ensure co-ordinated inter-sectoral action to address issues of food security and nutrition (Government of India, 2014a).

### 3.3 Food policy & the environment (SDG 12 responsible production & consumption, SDG 13 climate action)

A minority of food system policies specifically targeted the environment or the SDG 12, responsible production & consumption and SDG 13, climate action. Only 0.7% of the budget was allocated to almost a quarter (12/52) of the policies reviewed (₹1994 crores/US$251 million). These 12 policies were linked to the manually created climate budget area (i.e., National Action Plan on Climate Change), in the Ministry of Environment, Forests & Climate Change and the agricultural processing budget area (i.e., Pradhan Mantri Kisan SAMPADA Yojana), in the Ministry of Food Processing Industries. These two ministries received the smallest budgets of the food system relevant policies reviewed.

SAMPADA was one of the few policies reviewed which targeted food distribution and food processing activities (food processing was also referenced, to a small degree, in the dairy policies). This policy was mapped to SDG 12 responsible production & consumption in the Government of India NITI Aayog mapping exercise (Government of India, 2019a). The aim of the SAMPADA, Mega Food Parks policy is to increase efficiency, minimise waste, and maximise profit along the food supply chain by providing ‘modern infrastructure facilities’, such as cold food stores to support food processing and distribution. Minimising waste is relevant to the environment; however, the efficiency savings associated with this policy are also relevant to farmer livelihoods (farmer prosperity). If SAMPADA were to be included under section 3.1, then two fewer food system policies and ₹1081 fewer crores (US$142 million) would be considered relevant to the environment.

The ten policies identified relevant to SDG 13 climate action totalled ₹913 crores/US$120 million and represented 0.3% of the budget reviewed. This is significantly less than the funds associated with food system policies relevant to SDG 1 (24%), SDG 2 (51%), SDG 3 (21% or SDG 6 (3%). The National Action Plan on Climate Change and its sub-policies were included in this review as they highlight the impact of climate change on food production, citing predicted rising temperatures and variability in seasonal rains, as well as competition, for future arable land and irrigation resources between biofuels and food production. Two thirds of the budget for the National Action Plan is however, allocated to renewable energy policies (e.g., the National Solar Mission). These are indirectly linked to the food system, yet they were excluded from this review as no food reference was made in the policy documents.

Three of the National Action Plan on Climate Change policies (National Bamboo Mission, National Project on Agroforestry, National Project on Management of Soil Health and Fertility) were originally associated with the agricultural production budget. These three policies and their funds were manually added to the climate budget as sub-policies of the National Mission on Sustainable Agriculture, which in turn is a sub-mission of the National Action Plan on Climate Change. If these policies were reassigned back to the agriculture budget, then seven food system policies would be relevant to SDG 13 climate action, representing less than 0.2% of the budget and ₹452 crores/US$59 million.

### 3.4 Integration of environmental sustainability in Indian food policy

Environmental sustainability was integrated across approximately a third of the food system policies reviewed: 18/52 food policies included environmental sustainability into the framing of their policy documents. This equated to approximately a quarter of the total budget reviewed (₹79,943 crores/US$10.5 billion). Seven of these policies were part of the climate budget and seven were part of the agricultural production budget. The remaining policies were distributed over the rural employment, dairy, and clean India budget areas. There was no mention of environmental sustainability in any of the policies in the food subsidy, school meal, or social assistance budget areas; policies exclusively associated with food consumption.

The majority of policies that included environmental sustainability received an increase in funds across all three budget cycles (2018–2019, 2019–2020, 2020–2021). This was largely driven by increased funding for healthcare, rural employment, and agricultural production policies and might not have been specifically related to an environmental sustainability agenda. Funding for the directly relevant environmental sustainability policies (under the climate budget area) remained stable and consistently low across all three budget cycles.

In total, thirty-nine policies included the term ‘sustain’; however, 21 of these documents referred exclusively to the sustainability (continuation) of interventions rather than referencing environmental interventions or outcomes. Thirteen policies did not include a reference to sustainability of any kind. The agricultural processing budget area did not reference environmental sustainability, which supports the belief that this policy is more targeted to economic efficiency savings and does not currently integrate environmental sustainability into its policy framing.

The National Mission on Sustainable Agriculture was the main policy used to integrate sustainability into food policy. This a cross-ministerial policy, part of the National Action Plan on Climate Change in the Ministry of Environment, Forests & Climate Change, as well as largely implemented via a series of smaller policies in the Ministry of Agriculture and Farmer's Welfare. This mission was frequently referenced across policies in both ministries, indicating a degree of sustainability integration (e.g., quotes A & B)

“Mission on Integrated Development of Horticulture will work closely with National Mission on Sustainable Agriculture towards development of Micro-Irrigation for all horticulture crops and protected cultivation on farmers' field.”

Quote A, Agricultural production policy (Government of India, 2014b)

“Towards this end, National Mission for Sustainable Agriculture has been formulated for enhancing agricultural productivity especially in rainfed areas focusing on integrated farming, water use efficiency, soil health management and synergizing resource conservation.”

Quote B. Climate policy (Government of India, 2008)

The sustainable use of water and sustainable irrigation practices was frequently referenced. Eight of the 18 sustainable food system policies highlighted agricultural irrigation practices and the need to manage stressed water resources. It was not always clear from these references whether the management of vulnerable environmental resources was solely to protect future agricultural production and rural livelihoods, or if there was a broader agenda of environmental benefit (e.g., quotes C, D & E).

“The Himalayan ecosystem is vital to the ecological security of the Indian landmass, through providing forest cover, feeding perennial rivers that are the source of drinking water, irrigation, and hydropower, conserving biodiversity, providing a rich base for high value agriculture, and spectacular landscapes for sustainable tourism.”

Quote C, Climate policy (Government of India, 2008)

“For sustainability of the high productivity areas, special projects such as reclamation of problematic soils, development of water-logged areas and mitigation of adverse effect of climate change would be funded under the Mission for the promotion of National Food Security Mission crops of the district.”

Quote D, Agricultural production policy (Government of India, 2018c)

“Systematic identification and implementation of projects is highly recommended as it leads to creation of sustainable and productive assets for the community.” Quote 3,

Quote E, Rural employment policy (Government of India, 2013)

## 4 Discussion

A degree of environmental sustainability was integrated across the food policies reviewed, particularly in policies targeting food production. The policies that prioritised environmental sustainability were the ones that received the least funding. This indicates that the integration of environmental sustainability throughout food policy is in its infancy in India. These results are in line with previous research, which suggests low- and middle-income countries are less likely to prioritise environmental policies over those targeting economic growth or alleviation of poverty (Forestier and Kim, 2020). It is therefore, nor surprising that the majority of food policies reviewed in this study were aligned with SDG that represent long-standing policy goals in India (e.g., rural employment under SDG 1 no poverty; agricultural production growth and food and nutrition security under SDG 2 zero hunger & SDG 3 good health and well-being).

### 4.1 Disparate food policy

Study findings confirm the diverse role of food across a wide range of policies. Food policy was found to be disparate and fragmented, targeting different parts of the food system and involving a range of governmental ministries and departments. Some of these policies were directly relevant to the food supply chain, providing instruments to impact the production, distribution, consumption or disposal of food (e.g., the Public Distribution System). Other policies were linked to the wider food system e.g., the food security goals of the Annapurna social assistance scheme. This is not unique to India. For example, a recent work identified that food policy in England and South Africa continues to be developed and implemented in individual Ministries with a variety of different aims and objectives (Parsons et al., 2020; Pereira et al., 2020).

The range of food policies identified in this study provides a useful starting point for developing a holistic approach to food policy (Parsons and Hawkes, 2019b). Policies can only be co-ordinated, coherent, and integrated once the range of relevant food policies is understood and their complex inter-dependencies or in/direct conflict in priorities clearly established.

### 4.2 Increasing calls for sustainable food systems

Increasingly, national and international communities are recognising the false dichotomy of choosing between human and planetary requirements, with the realisation that we are reliant on the health of the environment for populations to prosper around the world. The FAO Committee on World Food Security, High Level Panel of Experts on Food Security and Nutrition recently released a consultation document integrating environmental sustainability into the definition of food security (FAO High Level Panel of Experts on Food Security & Nutrition, 2020b). This is to recognise the key role of the environment in promoting health for future generations. Furthermore, the European Commission produced a recent report titled ‘moving from food as a commodity to food as more of a common good’, with a central goal for policy to be developed and assessed in light of food sustainability in all its forms (European Commission, 2020).

The aspiration for sustainable food systems is evident in the policies reviewed in this study. The National Mission on Sustainable Agriculture in particular, as a sub-policy of the National Action Programme on Climate Change, provides a gateway to achieve cross-ministerial co-ordination and a degree of coherence and integration. By including sustainable food production into the framing of multiple policies the impact of the food system on the environment and likewise the impact of the environment on the food system is recognised. There is also evidence of political infrastructure to support sustainable policies in assigning NITI Aayog, as a cabinet level body, to act as a conduit for achieving the SDG and a way to monitor unintended consequences across SDG targets and universal goals.

### 4.3 Limitations

This scoping study is indicative of the food policies in India and not exhaustive. Study resources limited the review to central (national) policies. Individual states and union territories retain a degree of autonomy over the allocation and utilisation of financial funds due to the quasi-federal government structure in India. A review of state policies and budgets could have revealed a greater or lesser emphasis on environmental sustainability in relation to the geographical vulnerability to climate change within each state. Nor was every national policy relevant to the food system reviewed. This review did not capture new policies introduced outside the review period and several policies were excluded due to the search strategy employed. For example, not all policies related to fertiliser subsidies (Nutrient Based Subsidy), renewable energy policies (Grid Interactive Renewable Power) and financial credit schemes (the Pradhan Mantri Kisan Samman Nidhi, PM-KISAN) were included in the review. Specifically, the Pradhan Mantri Kisan Urja Suraksha evam Utthaan Mahabhiyan (KUSUM) policy to promote solar-powered irrigation was excluded due to its budget allocation of less than ₹500 crores/US$65.5 million in the 2020–2021 budget. In addition, the fishery (Blue Revolution) policies were excluded, as no funding was allocated to this area in the 2020–2021 budget. The use of alternative search terms to ‘food’, fewer exclusion criteria, and wider search sources (e.g., the use of key informant interviews), was considered unfeasible for the purpose of this review. A more inclusive review, however, would have provided an opportunity to establish the full range of policies that are in/directly related to food above and beyond those budgeted, which are heavily orientated towards subsidies.

Statutory bodies, which were not centrally sponsored schemes were also excluded from the review. Programme under the Food Safety and Standards Authority of India were not captured e.g., the plastic management consumer awareness campaign as part of the FSSAI ‘Eat Right India’ initiative (the FSSAI also received less than ₹500 crores/US$65.5 million in the 2020–2021 budget). Similarly, the 10.13039/501100003526Department of Agriculture Research & Education (10.13039/100006348DARE) plays an important role in crop sciences and agricultural education as an autonomous body rather than via centrally sponsored schemes.

Findings recognise the variety of ministries and departments responsible for different food policy missions, programme or schemes, yet aggregating small policies to their respective budget area might have underrepresented the variety of ministries involved in food policy that has been detailed elsewhere (Brown et al., 2020). Analysing the ministry and departmental responsibilities in more detail could have been insightful, in particular looking across political cycles. For example, the Ministry of Jal Shakti was created in 2019 to merge the Department of Drinking Water and Sanitation and the Department of Water Resources River Development and Ganga Rejuvenation. In addition, the Ministry of Animal Husbandry, Dairying and Fisheries was created in 2019 from a department of the same name, which used to reside under the Ministry of Agriculture and Farmers Welfare. The rearrangement of ministries and departments in itself can illustrate governmental priorities; however, this was not considered within the bounds of this study.

This review was designed to provide a broad overview of Indian food policies. The use of budget figures and content analysis was one route to establish the integration of environmental sustainability. There are opportunities for further research using alternative approaches, such as a more in-depth documentary analysis of individual policies or the use of key informant interviews. This could provide a more nuanced understanding of where and why environmental sustainability is or is not included in the framing of food policies in India e.g., in policy documents that pre-date the SDG, and do not use the words sustainable or sustainability. 10.13039/100014337Furthermore, in-depth analysis of a few select policies could identify and explore any direct conflicts between different food policies, such as between different subsidy programme from food production to consumption (e.g., the support of environmentally un/sustainable fertilisers, energy sources, eligible credit expenses, dairy/livestock/fishery production, food-based dietary guidelines etc.). This would be particularly useful for assessing the environmental focus of newly announced or redeveloped policies and how they integrate with the priorities of existing schemes. It would also be interesting to explore this for global food policy and formally analyse how individual policies might be in conflict, negate, or facilitate each other across all three dimensions of sustainability. This can contribute to establishing our progress as a global community in moving towards sustainable food systems (Davies, 2020).

## 5 Conclusions

The Indian commitment to global targets and the introduction of faciliatory infrastructure is noteworthy. As with all countries however, it is unlikely a complex problem such as sustainable food systems can be solved with limited budgetary funding or a lack of integrated policy. This study found that the policies which referenced sustainability received the least funding and a number of large food consumption policies made no reference to environmental sustainability. Results suggest an opportunity to support the integration of sustainability into policy across all parts of the food supply chain. In a country as geographically and socio-culturally diverse as India, this does not mean an aspiration for one sustainable diet, one sustainable farming practice, or one model of a sustainable food system. It does, however, provide a means for efficient policy making and risk management: to share data between ministries, recognise where existing policies may inhibit or facilitate each other, and adapt policy to changing (health, social, economic, political, environmental) priorities.

## Declaration of competing interest

The authors declare that they have no known competing financial interests or personal relationships that could have appeared to influence the work reported in this paper.
